# Supplementary material for: Identification of photoperiod-induced specific miRNAs in the adrenal glands of Sunite sheep (Ovis aries)
Source: Front Vet Sci. 2022 Jul 22;9:888207. doi: 10.3389/fvets.2022.888207 (PMC9354845; doi:10.3389/fvets.2022.888207)
Supplement: Supplementary file 1 [file Data_Sheet_1.ZIP › Supplementary file/miRDeep2 file.zip!/miRDeep2 file/Know_Structure/LP42A1/oar-mir-370.pdf]

miRBase precursor : oar-mir-370
Total read count : 6805
oar-miR-370-5p read count : 22
oar-miR-370-3p read count : 6776
remaining reads : 7

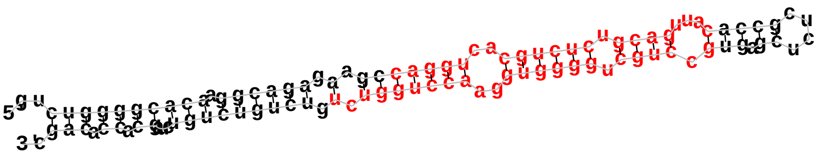

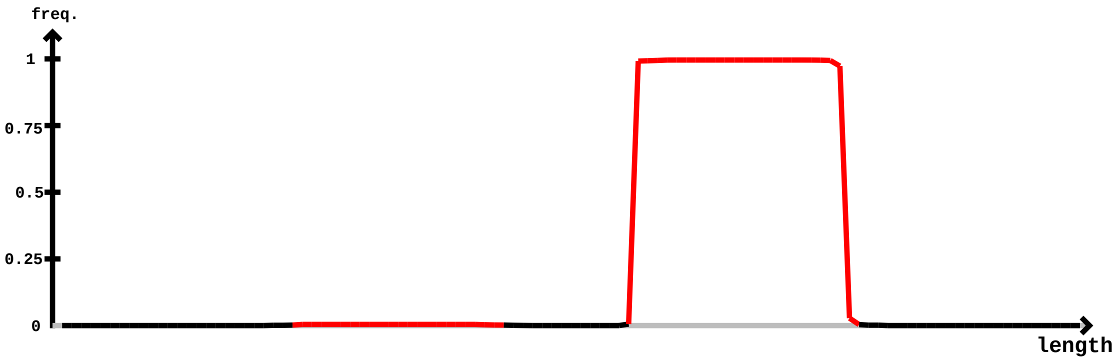


```
                                                                               oar-miR-370-3p
                        oar-miR-370-5p
5'- gucuggggcacaaggcagagaagccaggucacgucucugcaguuacaccgcucucgagugccugcugggguggaaccuggucugucugucugucuagcaccacagc -3'   exp
    ..((((((((((.((((((.(.(((((((..((((((.((((...(((((....)).))).)))).))))))..))))))).).)))))))))...)).)).))).    reads   mm   sample
    .....................agccaggucacgucucugcagu...............................................................    6       0    seq
    .....................agccaggucacgucucugcaguua.............................................................    1       0    seq
    .......................ccaggucacgucucugcagu...............................................................    1       0    seq
    .......................ccaggucacgucucugcaguu..............................................................    4       0    seq
    ........................caggucacgucucugcagu...............................................................    1       0    seq
    ........................caggucacgucucugcaguu..............................................................    2       0    seq
    ........................caggucacgucucugcaguua.............................................................    1       0    seq
    ........................caggucacgucucugcaguuac............................................................    7       0    seq
    ........................caggucacgucucugcaguuaca...........................................................    3       0    seq
    ........................caggucacgucucugcaguuacac..........................................................    3       0    seq
    ..........................................................ugccugcugggguggaaccug...........................    2       0    seq
    ..........................................................ugccugcugggguggaaccugg..........................    1       0    seq
    ..........................................................ugccugcugggguggaaccuggu.........................    36      0    seq
    ...........................................................gccugcugggguggaacc.............................    1       0    seq
    ...........................................................gccugcugggguggaaccu............................    3       0    seq
    ...........................................................gccugcugggguggaaccug...........................    6       0    seq
    ...........................................................gccugcugggguggaaccugg..........................    142     0    seq
    ...........................................................gccugcugggguggaaccuggu.........................    6391    0    seq
    ...........................................................gccugcugggguggaaccugguc........................    155     0    seq
    ...........................................................gccugcugggguggaaccuggucu.......................    12      0    seq
    ............................................................ccugcugggguggaaccuggu.........................    3       0    seq
    ............................................................ccugcugggguggaaccugguc........................    4       0    seq
    ............................................................ccugcugggguggaaccuggucu.......................    1       0    seq
    .............................................................cugcugggguggaaccuggu.........................    2       0    seq
    .............................................................cugcugggguggaaccugguc........................    5       0    seq
    .............................................................cugcugggguggaaccuggucu.......................    1       0    seq
    ..............................................................ugcugggguggaaccuggucu.......................    1       0    seq
    ..............................................................ugcugggguggaaccuggucugu.....................    9       0    seq
    ...............................................................gcugggguggaaccuggucugu.....................    1       0    seq
```
